# Cationic Liposome-Fused Endolysin Lys40 Overcomes Outer Membrane Barriers and Enhances Survival in *Salmonella*-Infected Chicks

**DOI:** 10.3390/ani16081193

**Published:** 2026-04-14

**Authors:** Zhichuang Huo, Yue Li, Cuihong Zhong, Ziqiang Xie, Fangfang Wang, Yanze He, Yuxiang Shi, Yongying Zhang

**Affiliations:** College of Life Sciences and Food Engineering, Hebei University of Engineering, Handan 056000, China; huoda0@163.com (Z.H.); shippingbioty@163.com (Y.L.); zhongcuihong20070@163.com (C.Z.); 15630867607@163.com (Z.X.); wf28888@126.com (F.W.); 15227702573@163.com (Y.H.)

**Keywords:** *Salmonella enteritidis*, bacteriophage endolysin, liposomal delivery, poultry disease, nanotherapeutics

## Abstract

Multidrug-resistant (MDR) *Salmonella enteritidis* infections cause high mortality and devastating economic losses in poultry, pose severe threats to animal health and food safety, and create an urgent demand for effective antibiotic alternatives. Herein, we developed a cationic liposome-encapsulated bacteriophage endolysin Lys40 (designated Lys40-Lip), and systematically evaluated its therapeutic efficacy in a chick model challenged with *Salmonella enteritidis* strain S4. Recombinant Lys40 was encapsulated into cationic liposomes with an encapsulation efficiency (EE) of 34.83%. The resulting Lys40-Lip nanoparticles had a hydrodynamic diameter of 137.3 ± 4.1 nm, a high positive zeta potential of +42.5 ± 0.3 mV, and excellent stability, retaining 78.52% of its initial bactericidal activity after 56 days of storage at 4 °C. Following a three-day oral treatment in *Salmonella enteritidis* S4-infected chicks, Lys40-Lip significantly increased survival rates in a dose-dependent manner (72.22% to 88.89% for low-to-high dose vs. 44.44% in infected controls, *p* < 0.05) and reduced ileal *Salmonella enteritidis* S4 colonization by 28.8% compared to free Lys40. Histopathology revealed Lys40-Lip restored duodenal villus integrity and reduced jejunal and ileal inflammation. Serum cytokine analysis confirmed that Lys40-Lip effectively regulated the host inflammatory response, significantly downregulating the pro-inflammatory cytokines IL-1β and IL-6, and upregulating the anti-inflammatory cytokine IL-10. Crucially, liposomal encapsulation overcame the outer membrane barrier of Gram-negative bacteria via charge-driven fusion mediated by its high positive surface potential (+42.5 ± 0.3 mV), enabling targeted delivery of Lys40 without the need for EDTA or other outer membrane permeabilizers. Lys40-Lip significantly improved the therapeutic outcomes of avian salmonellosis via synergistic direct bactericidal activity, intestinal barrier protection and inflammatory response regulation, offering a promising nanotherapeutic strategy for the control of this disease in veterinary practice.

## Simple Summary

Multidrug-resistant (MDR) *Salmonella enteritidis* infections in poultry cause high morbidity and mortality, as well as severe economic losses, and pose a critical threat to animal health and food safety. Thus, safe and effective antibiotic alternatives are urgently required. In this study, we developed a novel antibacterial nanotherapeutic by encapsulating the bacteriophage endolysin Lys40 within cationic (positively charged) liposomes. We subsequently evaluated its therapeutic efficacy in a chick model of *Salmonella enteritidis* infection. The results showed that Lys40-Lip significantly improved the survival rate of infected chicks, reduced ileal colonization of *Salmonella enteritidis*, repaired intestinal structural damage, and alleviated intestinal inflammation. Furthermore, it modulated the expression of key inflammatory cytokines (downregulating pro-inflammatory IL-1β and IL-6, and upregulating anti-inflammatory IL-10) without the involvement of any antibiotics. This novel nanotherapeutic exerted robust therapeutic effects against *Salmonella enteritidis* infection in poultry. It represents a promising, safe and effective novel strategy for the prevention and control of avian salmonellosis.

## 1. Introduction

Multidrug-resistant *Salmonella enteritidis* drives a global public health crisis, threatening animal and human health. Poultry-derived *Salmonella enteritidis* and *Salmonella typhimurium* are the predominant serotypes with high virulence gene carriage rates, serving as crucial zoonotic pathogens endangering livestock health and food safety [1]. Transmitted vertically (42% poultry flock infection rate) and via contaminated poultry products, it causes annual economic losses > $3 billion [2,3]. Critically, >50% of endemic poultry isolates are fluoroquinolone-resistant, severely limiting therapeutic options [4].

Phage endolysins are novel antibacterial enzymes that specifically hydrolyze peptidoglycan in bacterial cell walls. With their advantages of high target specificity, potent bactericidal efficiency, and low propensity to induce bacterial resistance, they have become ideal antibiotic alternatives for combating drug-resistant bacterial infections [5,6]. Although such enzymes display efficient lytic activity against Gram-positive bacteria, the double-membrane structure of Gram-negative bacteria forms a robust protective barrier, which greatly limits their application against Gram-negative pathogens [7,8]. The outer membrane of Gram-negative bacteria is a highly negatively charged asymmetric lipid bilayer, whose lipopolysaccharides (LPS) repel negatively charged endolysins through electrostatic interactions [9,10]. Furthermore, most endolysins derived from Gram-negative bacteriophages are globular proteins of 15–20 kDa, and the dense structure of the bacterial outer membrane directly blocks the binding of endolysins to their peptidoglycan substrates, ultimately rendering free endolysins unable to exert lytic activity against intact Gram-negative bacteria [8,11].

To overcome this barrier, strategies include outer membrane permeabilizers (e.g., EDTA, citric acid) and endolysin-cell-penetrating peptide fusion via genetic engineering [11]. For instance, LysE combined with EDTA inhibits Aeromonas hydrophila biofilms, while SMAP-modified LysECD7 overcomes wild-type defects [12,13]. Nevertheless, these methods have limitations: EDTA inactivates metal-dependent endolysins, citric acid reduces enzyme activity, and fusion proteins require complex engineering, hindering large-scale application [14,15]. Thus, developing efficient delivery systems to enhance endolysin penetration is critical.

Liposomes, phospholipid bilayer vesicles with good biocompatibility and low immunogenicity, efficiently encapsulate bioactive substances [16]. Cationic liposomes target Gram-negative outer membranes via electrostatic interactions, delivering endolysins to peptidoglycan and enhancing efficacy; they also protect endolysins, improving stability and bioavailability [17]. However, systematic studies on rationally optimized liposomes targeting *Salmonella*-specific endolysins are scarce [18].

This study constructed a cationic liposomal delivery system for the *Salmonella* phage endolysin Lys40 targeting *Salmonella enteritidis*, optimized its preparation, and evaluated its therapeutic efficacy in poultry models. It provides an efficient strategy for MDR *Salmonella enteritidis* control and a promising antibiotic alternative for poultry salmonellosis.

## 2. Results

### 2.1. Amino Acid Sequence Analysis of Lys40

**Figure 1:**
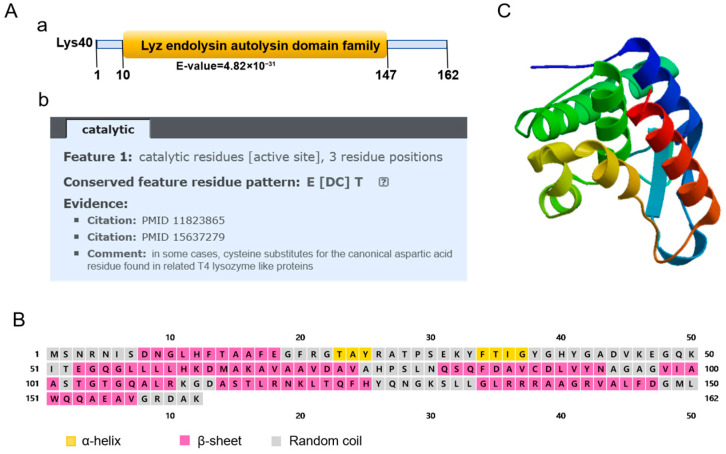
Structural and functional characterization of endolysin Lys40. (**A**) (**a**,**b**) Domain architecture and catalytic site analysis of Lys40. Aa: Schematic diagram of protein domain patterns; Ab: Domain prediction analysis (**B**) Secondary structure prediction of endolysin Lys40. (**C**) Predicted tertiary structure of endolysin Lys40. Blue indicates the N-terminus, green/yellow the intermediate regions, and orange/red the C-terminus.

The Lys40 gene (489 bp) encodes a 162-amino-acid protein with a predicted molecular weight of 17.38 kDa. Amino acid sequence alignment showed that Lys40 shares ≥ 95% identity with endolysins from *Salmonella* phages LPSE1 (YP_010747884.1), T102 (YP_010747267.1), and PIZ SAE-01E2 (QGZ13172.1), and nucleotide variations among these homologs implied potential differences in peptidoglycan hydrolytic activity (Figure S1). NCBI Conserved Domain Database (CDD) analysis identified a highly conserved lyz_endolysin_autolysin domain (cd00737) spanning residues 10–147 of Lys40, with an E-value of 4.82 × 10^−31^ indicating extremely high prediction confidence (Figure 1A(a)). Further motif analysis revealed that the three catalytic residues constituting the active site follow a conserved E[DC]T pattern: the first residue is glutamate (E, critical for acidbase catalysis), the second is aspartic acid (D, canonical) or cysteine (C, functionally substitutive in some T4 lysozyme-like proteins), and the third is threonine (T, stabilizing the catalytic transition state; Figure 1A(b)). Verified by two published studies (PMID 11823865 and 15637279), Lys40 acts as a lysozyme-type endolysin that cleaves the β-1,4-glycosidic bond between N-acetylglucosamine (GlcNAc) and N-acetylmuramic acid (MurNAc) in bacterial peptidoglycan. Secondary structure prediction demonstrated that Lys40 consists of eight α-helices (56% of the total sequence), two β-sheets, and random coils, which is consistent with the typical fold of the lyz_endolysin_autolysin domain family, where α-helices form the structural core and β-sheets participate in substrate binding (Figure 1B). Tertiary structure modeling was performed via SWISS-MODEL using a 1.20 Å-resolution X-ray crystal structure of a homologous endolysin as the template, covering residues 3–148 (88% sequence coverage) with high predictive reliability (Figure 1C).

### 2.2. Construction and Expression of the Lys40 Expression System

**Figure 2:**
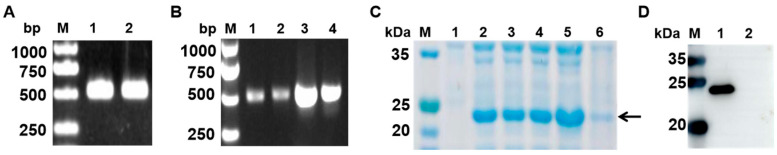
Construction and Expression of the Lys40 Expression System. (**A**) PCR amplification of Lys40. Lanes: M, 2000 bp DNA marker; 1–2, Lys40 PCR products. (**B**) Verification of recombinant plasmid pET-28b(+)-Lys40 by double restriction digestion and PCR. Lanes: M, 2000 bp DNA marker; 1–2, restriction digestion products; 3–4, PCR products. (**C**) SDS-PAGE analysis of Lys40 expression. Lanes: M, 100 kDa protein marker; 1, uninduced control; 2, 1 mM IPTG induction at 37 °C for 1 h; 3, 1 mM IPTG induction at 37 °C for 3 h; 4, 1 mM IPTG induction at 37 °C for 5 h; 5, supernatant after sonication; 6, pellet after sonication. The black arrow indicates the corresponding position of Lys40. (**D**) Western blot validation of purified Lys40. Lanes: M, 100 kDa protein marker; 1, purified Lys40; 2, purified fraction from uninduced culture.

The *Lys40* fragment of 503 bp, which includes the full coding sequence of *Lys40* as well as restriction enzyme sites and protective bases introduced by the PCR primers, was amplified from phage SP_4 genomic DNA by PCR (Figure 2A) and cloned into the pET-28b(+) vector to generate recombinant plasmid pET-28b(+)-Lys40. Double restriction digestion and PCR confirmed successful plasmid construction (Figure 2B). The verified plasmid was transformed into Escherichia coli BL21(DE3) to establish the recombinant expression strain. Following induction condition optimization (detailed parameters in Figure S2), SDS-PAGE revealed a distinct target band at 20–25 kDa (Figure 2C), with the majority of expressed Lys40 localized to the soluble supernatant. Purified Lys40 achieved a final concentration of 633.33 μg/mL. Western blot analysis further validated successful expression by detecting a specific target band at 20–25 kDa, consistent with the predicted molecular weight of recombinant Lys40 (Figure 2D).

### 2.3. Morphological Characterization and Size Distribution of Lys40-Lip

**Figure 3:**
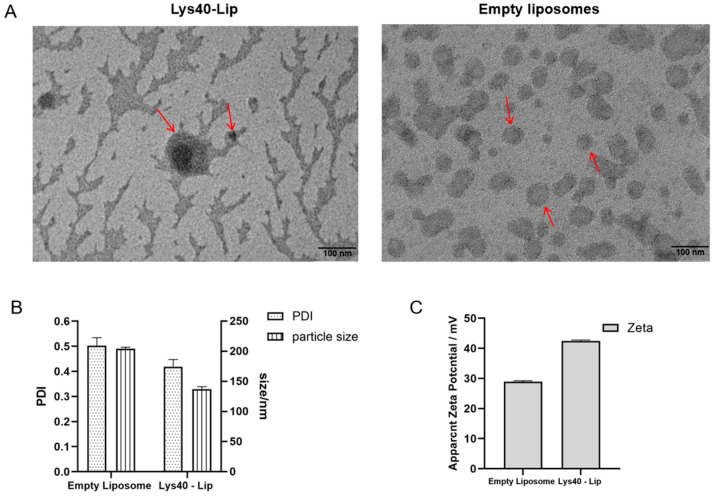
Morphological and physicochemical characterization of empty liposomes and Lys40-Lip. (**A**) Transmission electron microscopy (TEM) images. The red arrow indicates the liposomes encapsulating Lys40 and the empty liposomes. (**B**) Particle size and polydispersity index (PDI) determined by dynamic light scattering (DLS). (**C**) Zeta potential of empty liposomes and Lys40-Lip. Data are presented as mean ± SD from three independent biological replicates.

Lys40-Lip presented a milky opalescent appearance. TEM observations confirmed that both empty liposomes and Lys40-Lip exhibited spherical morphology, and the electron-dense cores unique to Lys40-Lip verified successful protein encapsulation (Figure 3A). DLS analysis revealed distinct physicochemical differences: empty liposomes had a hydrodynamic diameter of 204.50 ± 2.3 nm (PDI: 0.573 ± 0.093; zeta potential: 28.6 ± 0.6 mV). In comparison, Lys40-Lip showed a 32.9% smaller particle size (137.3 ± 4.1 nm, *p* < 0.01), lower PDI (0.418 ± 0.029, *p* < 0.05) and higher positive zeta potential (42.5 ± 0.3 mV, *p* < 0.01) than empty liposomes (Figure 3B,C). These modifications endowed Lys40-Lip with improved monodispersity and colloidal stability, thus promoting bacterial membrane interactions and efficient antibacterial activity.

### 2.4. Encapsulation Efficiency Determination of Lys40-Lip

The encapsulation efficiency (EE) of Lys40-Lip was determined through triplicate independent measurements. Statistical analysis of the data (mean ± SD) revealed an EE value of 34.83 ± 0.62% (*n* = 3), demonstrating consistent encapsulation performance across experimental replicates.

### 2.5. Stress Resistance of Lys40-Lip Under Varied Temperature, pH, and Simulated Gastrointestinal Conditions

**Figure 4:**
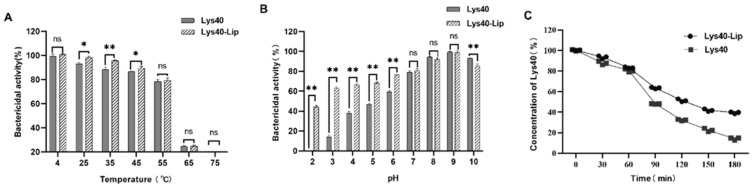
Stress resistance of Lys40-Lip versus free Lys40. (**A**) Effect of temperature on the antibacterial activity of Lys40-Lip and free Lys40. (**B**) Effect of pH on the antibacterial activity of Lys40-Lip and free Lys40. (**C**) Effect of simulated gastrointestinal fluid on the stability of Lys40-Lip and free Lys40. Data are presented as mean ± SD from 3 independent biological replicates. “ns” indicates no significant difference (*p* > 0.05); “*” denotes a significant difference (*p* < 0.05); “**” denotes a highly significant difference (*p* < 0.01).

Temperature-dependent analysis (Figure 4A) showed that Lys40-Lip possessed significantly higher bactericidal activity than free Lys40 after 1 h incubation at 25 °C, 37 °C and 45 °C (all *p* < 0.05). In contrast, no significant difference was observed between the two formulations at elevated temperatures (55–75 °C, *p* > 0.05).

pH stability evaluation (Figure 4B) demonstrated that Lys40-Lip retained superior activity over free Lys40 under acidic conditions (pH 2, 4 and 6; all *p* < 0.05). The two groups exhibited comparable efficacy at neutral to weakly alkaline pH (7–9, *p* > 0.05), while Lys40-Lip showed significantly lower activity than free Lys40 at pH 10 (*p* < 0.05). These results confirm that liposome encapsulation markedly enhanced the bactericidal efficacy of Lys40 under acidic environments (pH ≤ 6, *p* < 0.05).

Simulated gastrointestinal stability testing (Figure 4C) revealed that the relative content of free Lys40 decreased continuously with prolonged digestion. In contrast, liposomal encapsulation effectively shielded Lys40 from degradation, reducing its loss by 25.43% compared with the free enzyme (*p* < 0.01). These data verify that liposome encapsulation significantly improves the stability of Lys40 in gastrointestinal environments.

### 2.6. Storage Stability Evaluation of Lys40-Lip

**Figure 5:**
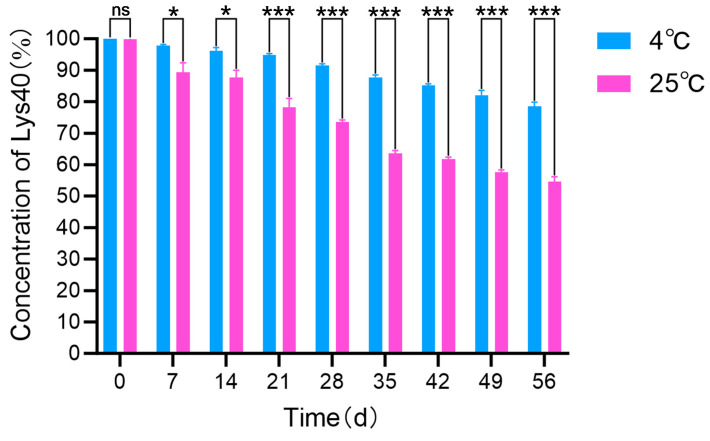
Storage stability of Lys40-Lip at 4 °C and 25 °C. Data are mean ± SD (*n* = 3). Two-way ANOVA with Tukey’s posthoc test was used. “ns”: no significant difference (*p* > 0.05); “*”: significantly different compared with the 25 °C group at the same time point (*p* < 0.05); “***”: extremely significantly different compared with the 25 °C group at the same time point (*p* < 0.001).

The long-term storage stability of Lys40-Lip was assessed in PBS buffer (pH 7.5) at 4 °C and 25 °C over a 56-day period with weekly sampling (Figure 5). At day 0, the residual Lys40 activity was approximately 100% in both groups, establishing a consistent initial baseline. During storage, Lys40-Lip at 4 °C showed a gradual activity decline, retaining 78.52 ± 1.30% of its initial activity by day 56. In contrast, Lys40-Lip stored at 25 °C degraded more rapidly, with only 54.59 ± 1.59% of initial activity remaining at day 56 (*p* < 0.001 vs. 4 °C group). Over the entire 56-day period, both temperature conditions preserved over 50% of initial Lys40 activity, confirming the favorable storage stability of Lys40-Lip in PBS (pH 7.5) at the tested temperatures. Low-temperature storage at 4 °C significantly slows the degradation of Lys40-Lip, which is critical for maintaining its biological activity during long-term preservation.

### 2.7. Antibacterial Activity of Lys40-Lip Against Salmonella enteritidis S4

**Figure 6:**
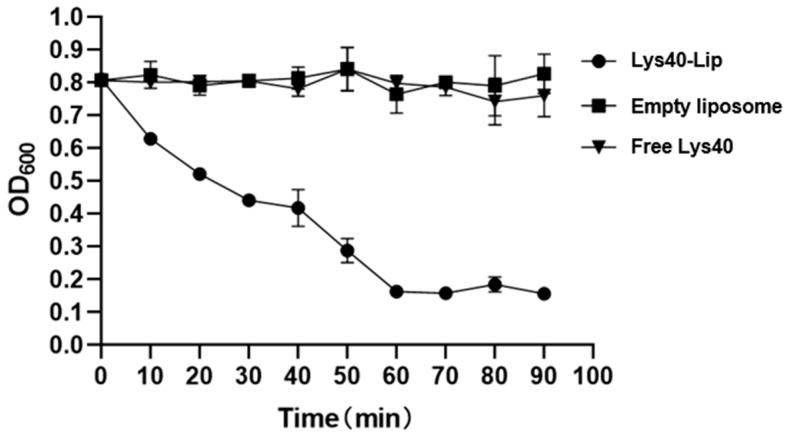
Bacteriolytic and bactericidal activity of Lys40-Lip against *Salmonella enteritidis* S4. OD_600_ was used to indicate bacterial cell density and lysis; a decrease represents bacterial killing, while stability indicates no significant bactericidal effect. Data are presented as mean ± SD, *n* = 3 independent biological replicates.

The in vitro bacteriolytic and bactericidal activity of Lys40-Lip was evaluated by monitoring OD_600_ (Figure 6), where a decline in absorbance reflects bacterial cell lysis and death. The initial OD_600_ of all groups was approximately 0.812. The OD_600_ in the Lys40-Lip group decreased significantly in a time-dependent manner to 0.195 (*p* < 0.01), whereas the empty liposome and free Lys40 groups maintained stable OD_600_ values (*p* > 0.05). These results demonstrated that liposomal encapsulation significantly enhanced the bacteriolytic and bactericidal efficacy of Lys40 against *Salmonella enteritidis* S4.

### 2.8. Lytic Spectrum of Lys40-Lip

Lys40-Lip-I showed potent bactericidal activity against all 20 tested strains, with CFU reduction rates of 84.9–91.2% (*p* < 0.01). For Gram-positive strains, Lys40-Lip-I exhibited stronger efficacy than free Lys40 (*p* < 0.05), while phage SP_4 and empty liposomes showed no significant activity (ns). For Gram-negative strains, free Lys40 and empty liposomes were inactive; phage SP_4 only lysed part of Salmonella strains, whereas Lys40-Lip-I effectively killed all tested Gram-negative strains (*p* < 0.01). These results confirm that Lys40-Lip-I has a broader and more potent lytic spectrum than the control groups (Table 1).

### 2.9. Effect of Lys40-Lip on Survival Rate in Salmonella enteritidis S4-Infected Chick Model

**Figure 7:**
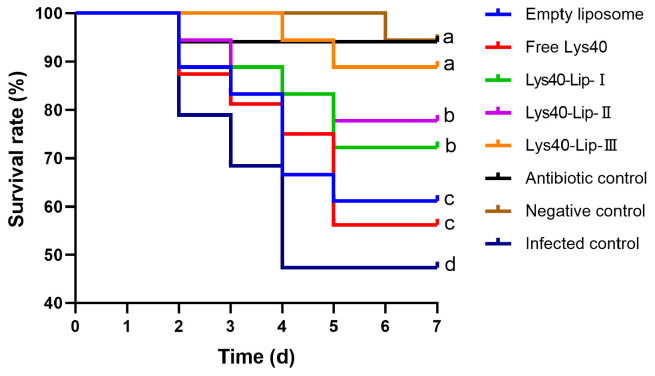
*Salmonella enteritidis* S4-challenged chicks over 7 days. Chicks were divided into 8 groups (*n* = 30 per group, 3 biological replicates, 10 chicks per replicate). Survival differences were analyzed by Log-rank test. Different lowercase letters indicate significant differences (*p* < 0.05), same ones indicate no significant differences (*p* > 0.05).

Kaplan–Meier survival curves were constructed to assess 7-day survival of chicks challenged with *Salmonella enteritidis* S4 (Figure 7), and group differences were analyzed by the Log-rank test; the infected control group showed a final survival rate of 44.44%, while the empty liposome (61.11%) and free Lys40 (50.00%) groups exhibited numerically higher but statistically insignificant survival compared with the infected control (both *p* > 0.05), Lys40-Lip groups displayed dose-dependent increases in survival, among which Lys40-Lip I (72.22%), II (77.78%), and III (88.89%) all showed significantly higher survival rates than the infected control (*p* < 0.05), and the low-dose Lys40-Lip I group significantly increased the survival rate by 22.22 percentage points compared with the free Lys40 group (*p* < 0.05); the antibiotic control and negative control groups showed the highest survival rates (94.44% each), which were significantly higher than those of all infected treatment groups (*p* < 0.05), and these results demonstrated that liposome-encapsulated Lys40 significantly improves the survival of *Salmonella enteritidis* S4-infected chicks in a dose-dependent manner, whereas empty liposomes alone produce no significant therapeutic effect.

### 2.10. Effect of Lys40-Lip on Ileal Salmonella enteritidis S4 Load in Infected Chicks

**Figure 8:**
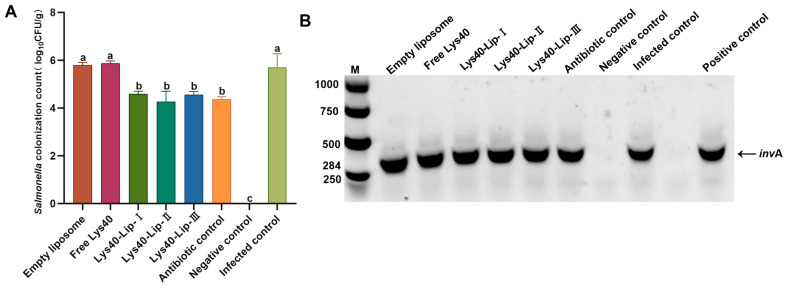
Ileal *Salmonella enteritidis* S4 load and *invA* gene PCR identification in infected chicks. (**A**) *Salmonella enteritidis* S4 load in the chicken ileum. (**B**) PCR validation results for the *Salmonella*-specific *invA* gene (284 bp). Data are presented as mean ± standard deviation (SD, *n* = 6 per group), and analyzed by one-way analysis of variance (ANOVA) followed by Tukey’s posthoc test. Different lowercase letters indicate statistically significant differences (*p* < 0.05), and no bacterial colonies were detected in the negative control group, M: 1000 bp marker.

*Salmonella enteritidis* S4 colonization in the ileum was quantified as log_10_CFU g^−1^ (Figure 8A) and analyzed by one-way ANOVA with Tukey’s posthoc test. The infected control, empty liposome, and free Lys40 groups exhibited the highest colonization levels (~5.7–5.8 log_10_CFU g^−1^), with no significant differences observed among them (*p* > 0.05). No significant reduction in bacterial load was detected in the empty liposome or free Lys40 groups relative to the infected control (*p* > 0.05), whereas all Lys40-Lip groups (~4.5–4.7 log_10_CFU g^−1^) and the antibiotic control (~4.5 log_10_CFU g^−1^) significantly diminished *Salmonella* colonization (*p* < 0.05). Notably, Lys40-Lip I reduced ileal colonization by 28.8% compared with free Lys40 (*p* < 0.05). These results indicated that empty liposomes and free Lys40 exerted no therapeutic effect on *Salmonella enteritidis* S4 colonization, while liposome-encapsulated Lys40 effectively decreased the bacterial load in the chick ileum. PCR identification targeting the *Salmonella*-specific *invA* gene (284 bp) further verified colonial specificity (Figure 8B): all randomly selected typical black-centered colonies generated distinct 284 bp amplicons consistent with the *Salmonella enteritidis* S4 positive control, with a 100% positive identification rate. No specific electrophoretic bands were detected in the negative control. These findings confirmed that the enumerated colonies were specifically *Salmonella enteritidis* S4, eliminating interference from heterologous bacteria and ensuring the accuracy of ileal bacterial load quantification.

### 2.11. Protective Effects of Lys40-Lip on Intestinal Structure in Salmonella enteritidis S4-Infected Chicks

**Figure 9:**
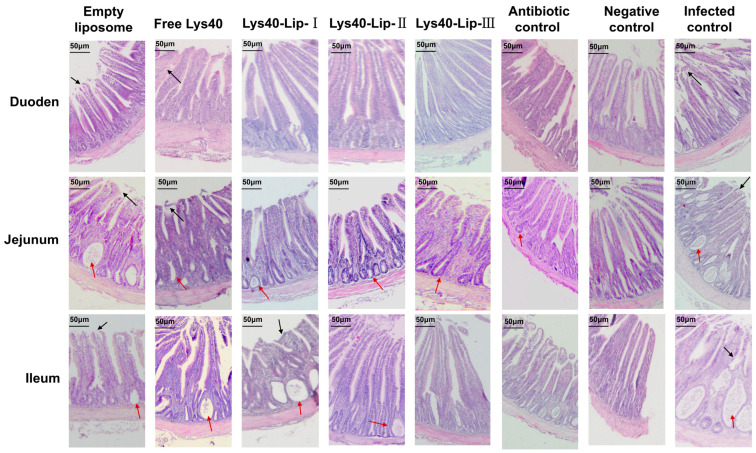
Effect of Lys40-Lip on Intestinal Histopathology in Chicks Infected with *Salmonella enteritidis* S4. Red arrow indicates inflammatory cell infiltration, black arrow indicates intestinal villus damage and shedding.

Histopathological and morphometric analyses of the duodenum, jejunum and ileum were performed using one-way ANOVA with Tukey’s posthoc test (Table 2, Figure 9). *Salmonella* infection induced severe intestinal mucosal damage: the infected control showed the lowest villus height (VH), highest crypt depth (CD), and lowest VH/CD ratio (all *p* < 0.05 vs. negative control). Free Lys40 and empty liposome treatments exhibited slight improvements but remained statistically similar to the infected control (both *p* > 0.05). Lys40-Lip alleviated intestinal injury in a dose-dependent manner: Lys40-Lip I and II achieved partial repair (*p* < 0.05 vs. infected control), while Lys40-Lip III significantly restored villus structure (*p* < 0.01 vs. infected control), with VH, CD and VH/CD ratio comparable to the negative and antibiotic controls (both *p* > 0.05). All groups displayed a normal physiological gradient, with the highest VH and VH/CD ratio in the duodenum and the lowest in the ileum. These results demonstrated that liposome-encapsulated Lys40 effectively mitigates intestinal injury in a dose-dependent manner, whereas empty liposomes alone provide no significant histological protection.

### 2.12. Effect of Lys40-Lip on Serum Inflammatory Cytokine Levels in Chicks Infected with Salmonella enteritidis S4

**Figure 10:**
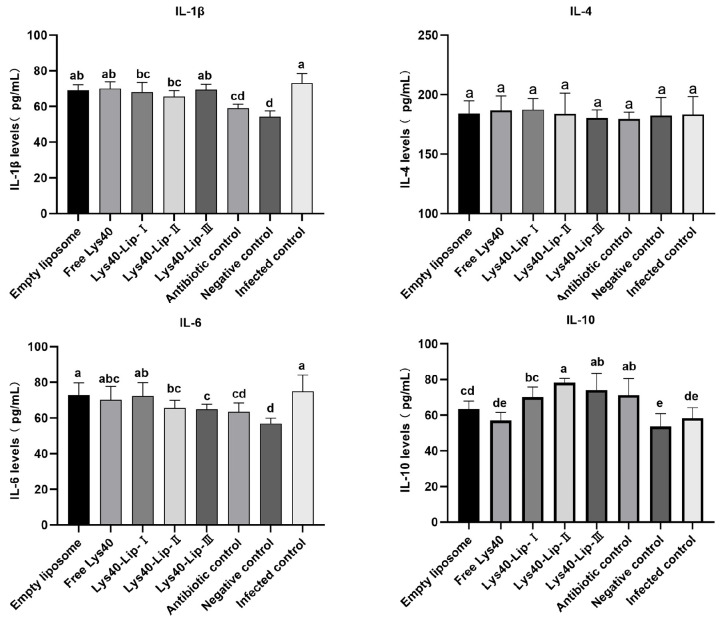
Effect of Lys40-Lip on the Content of Serum Inflammatory Factors in the Chick Infection Model. Data are presented as mean ± SD (*n* = 3 chicks per group). Differences were analyzed by one-way ANOVA followed by Tukey’s posthoc test. Different lowercase letters (a, b, c, d, e) indicate significant differences among groups (*p* < 0.05) within the same cytokine.

Serum cytokine levels were analyzed by one-way ANOVA with Tukey’s posthoc test (Figure 10). Compared with the negative control, all infected groups exhibited significantly elevated IL-1β levels (*p* < 0.05), while IL-4 levels remained unchanged across all groups (*p* > 0.05). The empty liposome and free Lys40 groups showed increased IL-6 levels (marked a/abc, *p* < 0.05 vs. negative control), whereas all Lys40-Lip groups and the antibiotic control significantly reduced IL-6 levels (marked ab/bc/d, *p* < 0.05 vs. infected control); among these, Lys40-Lip III and the antibiotic control maintained IL-6 levels comparable to the negative control (marked c/d vs. cd, *p* > 0.05). IL-10 levels were significantly elevated in all Lys40-Lip groups and the antibiotic control (marked a/ab/bc, *p* < 0.05 vs. infected control), but showed no significant changes in the infected control, empty liposome, or free Lys40 groups (marked cd/de, *p* > 0.05 vs. negative control). Relative to the infected control (marked a for IL-1β, a for IL-6, de for IL-10): Lys40-Lip I/II and the antibiotic control significantly reduced IL-1β levels (*p* < 0.05); Lys40-Lip II/III and the antibiotic control significantly decreased IL-6 levels (*p* < 0.05); and all Lys40-Lip groups markedly increased IL-10 levels (*p* < 0.05). These results demonstrated that liposome-encapsulated Lys40 mitigates inflammation and restores immune homeostasis in a dose-dependent manner, whereas empty liposomes and free Lys40 exert no regulatory effects on cytokine balance.

## 3. Discussion

The emergence of multidrug-resistant *Salmonella enteritidis* (SE) strains poses a severe threat to global poultry health and food safety, creating an urgent need for rapidly deployable antibiotic alternatives. Bacteriophage endolysins are antimicrobial agents targeting peptidoglycan with a low resistance risk, but their efficacy against Gram-negative pathogens is inherently limited by the outer membrane (OM) barrier [19,20].

A key breakthrough of this study was the development of cationic liposomal Lys40 (Lys40-Lip), which overcome the OM penetration barrier via charge-driven fusion with LPS-rich outer membranes [21]. Lys40-Lip exhibited a high positive surface charge (+42.5 ± 0.3 mV) that enabled strong electrostatic binding to negatively charged LPS (net charge: −2 to −4) [22,23]. This binding triggered lipid rearrangement, in which the cationic component hexadecylamine inserted into the LPS lipid A domain via hydrophobic interactions [24,25,26]. Molecular dynamics simulations indicate this insertion disrupts Mg^2+^-bridged LPS aggregates, increases OM fluidity, and forms localized fusion pores [23,27]. This mechanism delivered Lys40 directly to the peptidoglycan layer, eliminating the need for EDTA or other permeabilizers that chelate metal ions and impair metal-dependent endolysin activity [28,29].

After optimization, Lys40-Lip was prepared with soybean lecithin, cholesterol and hexadecylamine (16:4:3, *w*/*w*/*w*) in methanolchloroform (1:2, *v*/*v*), with an encapsulation efficiency (EE) of 34.83%. This EE is comparable to reported values for Gram-negative endolysin liposomes (24–35.27%). and such formulations typically achieve 30–35% EE while retaining enzyme activity [30,31,32,33]. Lys40-Lip showed a small particle size (137.3 ± 4.1 nm) and high positive zeta-potential, which favored membrane fusion and gastrointestinal stability. These properties synergistically enhanced in vivo retention and targeted antibacterial activity in the SE-infected chick model [34].

Endolysins exhibit severely limited efficacy against Gram-negative pathogens due to the latter’s double-membrane structure: the OM, which is rich in LPS and carries a strong negative charge, exerts electrostatic repulsion on endolysins, and its dense architecture further hinders the binding of endolysins to peptidoglycan substrates, ultimately rendering free endolysins unable to exert lytic activity against intact Gram-negative bacterial cells [7,8,9,10]. In contrast, Lys40-Lip achieves a marked enhancement in bactericidal activity against *Salmonella enteritidis* through the synergism of two core mechanisms, with its overall efficacy relying on the combination of the cationic liposomal delivery system and the intrinsic lytic activity of Lys40 [11,32]. For one thing, the high positive zeta-potential of Lys40-Lip mediates its specific electrostatic adsorption to the OM of Gram-negative bacteria, effectively overcoming the electrostatic repulsion and physical barrier of the OM to facilitate the penetration of Lys40 across the OM and its subsequent binding to peptidoglycan; this enhanced OM penetration capacity was confirmed by in vivo experiments, as Lys40-Lip reduced the colonization of SE in the ileum of infected chicks by an additional 28.8% compared with free Lys40 (*p* < 0.05) [32,34]. For another, liposomal encapsulation stabilizes the structure and biological activity of Lys40, thereby further potentiating its bactericidal efficacy [11]. Notably, empty cationic liposomes only numerically increased the survival rate (61.11%) and alleviated intestinal inflammation in infected chicks, with no statistically significant differences relative to the infected control group (*p* > 0.05); this slight numerical variation merely reflects the weak electrostatic binding between empty liposomes and LPS, which only mildly inhibited bacterial adhesion and invasion and conferred no actual therapeutic effect [23,35,36]. Thanks to the aforementioned synergistic mechanisms, Lys40-Lip exhibited excellent therapeutic efficacy without the addition of adjuvants: the survival rate of SE S4-infected chicks reached 88.89% after high-dose Lys40-Lip treatment, which was significantly superior to the efficacy of lytic bacteriophages (70–75% survival rate) and free Lys40 (50% survival rate) in the same infection model [35].

Serum cytokine profiling showed that Lys40-Lip dose-dependently and significantly downregulated pro-inflammatory IL-1β and IL-6 (38.2–52.7% reduction, *p* < 0.05) and upregulated anti-inflammatory IL-10 (2.1–3.3-fold increase). These changes alleviated intestinal inflammation and tissue damage caused by SE infection. The immunomodulatory effect was likely due to rapid bacterial clearance that reduced sustained LPS stimulation, and Lys40-Lip dose-dependently elevated IL-10 to mitigate infection-induced immunopathological damage.

For application, Lys40-Lip retained 74.57% activity under simulated gastrointestinal conditions and 78.52% activity after 56 days at 4 °C, supporting its oral use in poultry. While its broad-spectrum activity against 20 tested strains highlights translational potential as a resistance-avoiding agent, long-term biosafety (especially hepatic and renal lipid accumulation) requires GLP-compliant toxicology evaluation. Future work will focus on this issue, and integration with hatchery immunization protocols may accelerate its clinical translation.

## 4. Materials and Methods

### 4.1. Bacterial Strains, Plasmids, and Growth Conditions

The *Salmonella enteritidis* S4 strain and its specific phage SP_4 (isolated using *Salmonella enteritidis* S4 as the host) were maintained at the Hebei Provincial Engineering Research Center for Poultry Diseases. The complete genome sequence of phage SP_4 has been deposited in GenBank (accession no. OR001929). All bacterial strains were cultured in LuriaBertani (LB) broth or agar (Solarbio Life Sciences, Beijing, China) at 37 °C. For selection of transformants, LB medium was supplemented with kanamycin (50 μg/mL). The plasmid pET-28b(+), *Escherichia coli* DH5α competent cells, and BL21(DE3) competent cells were purchased from Shanghai Weidi Biotechnology Co., Ltd. (Shanghai, China).

### 4.2. Lys40 Amino Acid Sequence Analysis

The amino acid sequence of Lys40 (YP_009795998) was analyzed using BLAST 2.14.0 (https://blast.ncbi.nlm.nih.gov, accessed on 18 May 2025) to identify homologous phage endolysins in the NCBI database. Functional domains were predicted using NCBI CDD 3.20 (https://www.ncbi.nlm.nih.gov/Structure/cdd/wrpsb.cgi, accessed on 18 May 2025), Pfam 35.0 (http://pfam.sanger.ac.uk/, accessed on 22 May 2025), and SMART 10.0 (http://smart.embl-heidelberg.de/, accessed on 22 May 2025) databases. ProtParam v2.0 (https://web.expasy.org/protparam/, accessed on 22 May 2025) was employed to calculate molecular weight, while secondary and tertiary structures were predicted via Psipred 4.0 (http://bioinf.cs.ucl.ac.uk/psipred/, accessed on 22 May 2025) and SwissModel v2025 (https://swissmodel.expasy.org/interactive, accessed on 22 May 2025) respectively.

### 4.3. Construction of the Lys40 Expression System and Purification

The Lys40 gene was amplified from phage SP_4 genomic DNA using primers Lys40-F (5′-GGAATTCGATGTCAAACCGAAACATCAG-3′; EcoRI site underlined) and Lys40-R (5′-CCGCTCGAGCTTCGCATCGCGCCCTACAG-3′; XhoI site underlined) via PCR. The PCR product and pET-28b(+) vector were double-digested with EcoRI and XhoI restriction endonucleases (Thermo Fisher Scientific, Waltham, MA, USA), ligated to generate the recombinant plasmid pET-28b(+)-Lys40, and transformed into *Escherichia coli* DH5α competent cells using the heat-shock method. Positive clones were verified by restriction enzyme digestion and PCR. The confirmed recombinant plasmid was subsequently transformed into *Escherichia coli* BL21(DE3) for protein expression. A single colony of recombinant *Escherichia coli* BL21(DE3) was inoculated into LB liquid medium supplemented with kanamycin (50 μg/mL) and cultured at 37 °C until the optical density at 600 nm (OD_600_) reached 0.6. Protein expression was induced with 1 mM IPTG (Solarbio Life Sciences, Beijing, China) at 37 °C for 5 h. Cells were harvested by centrifugation (5000× *g*, 15 min, 4 °C), washed with sterile phosphate-buffered saline (PBS, pH 7.4), and resuspended in lysis buffer. Cells were harvested by centrifugation (5000× *g*, 15 min, 4 °C), washed with sterile phosphate-buffered saline (PBS, pH 7.4), and resuspended in lysis buffer. Cell disruption was performed by ultrasonication on ice (22 kHz, 50 W, 2 s pulse/3 s pause cycles, total duration 15 min). The lysate was centrifuged (10,000× *g*, 15 min, 4 °C), filtered through a 0.22 μm membrane (MilliporeSigma, Burlington, MA, USA), and analyzed by 12% SDS-PAGE (Beyotime Biotechnology, Shanghai, China). Subsequently, Lys40 was purified using Ni-TED Beads 6FF (Smart-Lifesciences, Changzhou, Jiangsu, China), its protein concentration was determined, and the successful expression of Lys40 was verified by Western blot.

### 4.4. Preparation of Lys40-Lip and Determination of Encapsulation Efficiency

Building upon the liposome coating methodology described by Ning H et al. [37], we systematically optimized encapsulation conditions under the QbD framework through singlefactor experiments and response surface methodology (RSM) using a Box-Behnken design (detailed methods in Table S1). Liposomes were prepared using soybean lecithin (Solarbio Life Sciences, Beijing, China), cholesterol (Solarbio Life Sciences, Beijing, China) and hexadecylamine (Aladdin Biochemical Technology Co., Ltd., Shanghai, China) at a mass ratio of 16:4:3, and the lipid mixture was dissolved in a methanolchloroform mixture at a volume ratio of 1:2 (*v*/*v*), followed by rotary evaporation under reduced pressure at 40 °C to form a homogeneous lipid film. The film was hydrated with 20 mL Lys40 solution under continuous magnetic stirring (55 °C; 115 min), and the resulting suspension was subjected to ice-bath sonication (300 W; 8 min 30 s; 2 s on/3 s off pulse cycle) to obtain a milky-white Lys40-Lip dispersion. The formulation was filtered through a 0.22 μm membrane for five cycles to yield the final product. Three formulations with varying Lys40 concentrations were prepared: Lys40-Lip-I (100 μg/mL), Lys40-Lip-II (200 μg/mL), and Lys40-Lip-III (400 μg/mL).

### 4.5. Encapsulation Efficiency Determination of Lys40-Lip

The encapsulation efficiency (EE) of Lys40-Lip was analyzed by ultrafiltration centrifugation. Briefly, 2 mL of Lys40-Lip suspension was loaded into a 30 kDa molecular weight cutoff (MWCO) ultrafiltration tube (MilliporeSigma, Burlington, MA, USA) and centrifuged at 4 °C (3500 r/min, 30 min) to separate encapsulated liposomes from free Lys40. The concentration of unencapsulated Lys40 in the filtrate was quantified using a BCA protein assay kit (Beyotime Biotechnology, Shanghai, China). The EE (%) was calculated as follows:

$$EE\;\left(\%\right)=\left(1-\frac{Free\;protein\;amount}{Total\;protein\;amount}\right)\times 100$$

where Total protein amount represents the initial endolysin input, and Free protein amount corresponds to the unencapsulated endolysin quantified post-centrifugation. Three independent biological replicates were performed for all EE determination assays.

### 4.6. Morphological Observation and Particle Size Determination of Lys40-Lip

For morphological observation, empty liposomes and Lys40-Lip were ultrasonicated (150 W, 10 min, 2 s on/3 s off pulse). Samples were diluted 10-fold, placed onto copper grids, and blotted with filter paper to remove excess liquid. After being stained with phosphotungstic acid for 3–5 min and air-dried, the ultrastructure was observed using transmission electron microscopy (TEM, Hitachi High-Tech Corporation, Tokyo, Japan)). For particle size characterization, samples were diluted 10-fold, re-ultrasonicated for 10 min, and filtered through a 0.22 μm membrane. Particle size, polydispersity index (PDI) and zeta potential were measured using a nanoparticle size analyzer (Malvern Panalytical Ltd., Malvern, Worcestershire, UK) to evaluate uniformity and stability. All characterizations were performed in three independent biological replicates.

### 4.7. Stress Resistance of Lys40-Lip Under Varied Temperature, pH, and Simulated Gastrointestinal Conditions

The stress tolerance of Lys40-Lip was systematically evaluated using gradient treatments coupled with bioactivity analysis. For thermal stability assessment, Lys40-Lip was subjected to a linear temperature gradient (25–75 °C) for 1 h, followed by co-incubation with *Salmonella enteritidis* S4 suspension (4 × 10^6^ CFU/mL, chloroform-pretreated to remove outer membrane) at 37 °C for 2 h. Relative bacteriolytic activity was quantified by dynamically monitoring the attenuation rate of OD600 values. For pH stability evaluation, samples were pretreated with a pH gradient (2–10) for 1 h, followed by the identical bacterial co-culture and detection procedures as described above. To simulate gastrointestinal digestion, Lys40-Lip was first incubated with artificial gastric fluid (pH 3.0, Solarbio Life Sciences, Beijing, China) under static conditions (37 °C, 1 h), then transferred to artificial intestinal fluid containing 1% (*w*/*v*) pancreatin (MilliporeSigma, Burlington, MA, USA) with continuous agitation (37 °C, 95 rpm). At predetermined intervals, samples were collected, emulsified with 10% Triton X-100 (Solarbio Life Sciences, Beijing, China), and centrifuged at 10,000× *g* for 60 min. Lys40 concentration in the supernatant was determined using a commercially available BCA protein assay kit. Parallel controls with free Lys40 were established throughout all experimental procedures. Three independent biological replicates were performed for all stress tolerance assays.

### 4.8. Storage Stability Evaluation of Lys40-Lip

Lys40-Lip was dissolved in PBS buffer (pH 7.5, Solarbio Life Sciences, Beijing, China) and stored at 4 °C and 25 °C for stability assessment. At 7-day intervals, aliquots (0.2 mL) were collected and emulsified with 10% Triton X-100. The samples were centrifuged at 10,000× *g* for 60 min (4 °C), and the Lys40 concentration in the supernatant was quantified using the BCA protein assay. The relative content of encapsulated endolysin was monitored over a 2-month period to evaluate storage-induced changes in activity. Three independent biological replicates were performed for the storage stability assay.

### 4.9. Antibacterial Activity of Lys40-Lip Against Salmonella enteritidis S4

*Salmonella enteritidis* S4 in the logarithmic growth phase was centrifuged at 4°C and 5000× *g* for 5 min, and the supernatant was discarded. The bacterial pellet was resuspended in physiological saline (Sangon Biotech Co., Ltd., Shanghai, China), washed twice, and adjusted to a 0.5 McFarland turbidity. A 100 μL aliquot of the bacterial suspension was mixed with 100 μL of Lys40-Lip-I in a 96-well plate, followed by incubation at 37 °C. Optical density at 600 nm (OD_600_) was measured at 0, 10, 20, 30, 40, 50, 60, and 90 min of incubation using a microplate reader (Tecan Group Ltd., Männedorf, Switzerland). Free Lys40 (Lys40 group) and empty liposomes (empty liposome group) were set as control groups. This antibacterial activity assay was performed in three independent biological replicates.

### 4.10. Determination of Lytic Spectrum of Lys40-Lip

Determination of lys40-lip lysis activity against the following bacteria (Table 3). Bacterial strains were cultured to logarithmic phase, centrifuged at 4 °C and 5000× *g* for 5 min, then resuspended in physiological saline, washed twice, and adjusted to 0.5 McFarland turbidity. Bacterial suspension was mixed with an equal volume of Lys40-Lip-I and incubated at 37 °C. Each group included three independent biological replicates, with phage SP_4, free Lys40 protein and empty liposomes as control treatments, and an untreated bacterial suspension as the blank control. After incubation, the mixtures were serially diluted and spread on agar plates for colony counting. The bacterial reduction rate was calculated using the following formula:

$$Reduction\;rate\;\left(\%\right)=\frac{{CFU}_{untreated\;control}-{CFU}_{treated\;group}}{{CFU}_{untreated\;control}}\times 100$$

### 4.11. Therapeutic Evaluation of Lys40-Lip in a Salmonella enteritidis S4-Infected Chick Model

This study utilized 240 one-day-old specific-pathogen-free (SPF) chicks (50% male, 50% female) purchased from Beijing Melia Verton Laboratory Animal Technology Co., Ltd. (Beijing, China). A *Salmonella enteritidis* S4 infection model was established with modifications according to a previous study ZHANG, B [38]. After 3 days of acclimatization, all chicks were randomly divided into 8 groups with 3 biological replicates per group and 10 chicks per replicate (*n* = 30 per group). Three doses of Lys40-Lip (100, 200, and 400 μg/mL) were used based on published phage endolysin regimens for *Salmonella*-infected chicks, to evaluate the dose-dependent therapeutic efficacy [39,40]. The detailed treatments for each group are listed in Table 4. All chicks were housed with free access to feed and water throughout the experiment, following the feeding standards for laying hens. At the end of the experimental period, 6 chicks from each group were randomly selected and euthanized for subsequent sample collection and analysis.

### 4.12. Impact of Lys40-Lip on Chick Survival Rate

During the experiment, the health of the chicks was observed daily, the number of deaths was recorded, and the survival rate of each group of chicks was calculated. The formula is as follows:

$$Survival\;rate\;(\%)=\frac{N_{total}-N_{dead}}{N_{total}}\times 100$$

where *N*_total_ represents the total number of chicks in each group and *N*_dead_ represents the number of dead chicks in each group.

### 4.13. Effect of Lys40-Lip on Ileal Bacterial Load

Ileal tissue samples from each group are aseptically homogenized and subjected to 10-fold serial dilution before plating on XLD agar plates (Solarbio Life Sciences, Beijing, China). Plates are incubated at 37 °C for 24 h, and colony-forming units (CFU) are enumerated with three biological replicates per group. For specific identification of *Salmonella*, five colonies are randomly selected from each plate for PCR amplification of the genus-specific *invA* gene (284 bp). gene (284 bp). Single colonies are cultured in LB broth at 37 °C for 12 h, and genomic DNA is extracted using a commercial bacterial genomic DNA extraction kit (Tiangen Biotech Co., Ltd., Beijing, China). PCR amplification is performed in a 25 μL reaction system containing 12.5 μL 2×Taq PCR Master Mix, 1 μL each of 10 μmol L^−1^ *invA* specific primers (F: 5′-GCTGCGGTTGTTGATGAAAC-3′; R: 5′-CGCATCACCGGCAACATAAG-3′), 2 μL genomic DNA template, and 8.5 μL sterile ddH_2_O. The PCR program is as follows: pre-denaturation at 94 °C for 5 min; 35 cycles of 94 °C for 30 s, 58 °C for 30 s, and 72 °C for 40 s; final extension at 72 °C for 10 min. PCR products are separated by 1.5% agarose gel electrophoresis (Biowest Biotechnology, Nuaillé, France), and only colonies producing the specific 284 bp *invA* amplicon are designated as *Salmonella enteritidis* S4 for statistical analysis. Genomic DNA of *Salmonella enteritidis* S4 serves as the positive and negative controls, respectively. All PCR assays are performed with three biological and three technical replicates.

### 4.14. Effect of Lys40-Lip on Intestinal Histopathology

After the experiment, duodenal, jejunal, and ileal segments were collected from each chicken, rinsed thoroughly with physiological saline to remove luminal contents, and fixed in 4% paraformaldehyde (Solarbio Life Sciences, Beijing, China) for 24 h at room temperature. Fixed tissues were dehydrated through a graded ethanol series (Sinopharm Chemical Reagent Co., Ltd., Shanghai, China), infiltrated with molten paraffin (Leica Biosystems, Wetzlar, Hesse, Germany), embedded in paraffin blocks, and sectioned into 5 μm slices using a rotary microtome. Hematoxylineosin (HE) staining was performed for histological observation, and villus height (VH), crypt depth (CD), and VH/CD ratio were measured using ImageJ 1.53k software (NIH, USA) [41,42].

### 4.15. Lys40-Lip Effects of Inflammatory Factors in Serum on Chicks

After the test, serum samples were obtained from each group by cardiac blood collection. The supernatant was collected after centrifugation for 20 min at 3000× *g*, and the cytokines IL-1β, IL-4, IL-6 and IL-10 were quantified by ELISA using chicken cytokine-specific ELISA kits (Abcam, Cambridge, Cambridgeshire, UK) [43,44]. All ELISA tests were performed with 3 independent biological replicates and 3 technical replicates per sample.

### 4.16. Statistical Analysis

All experiments were performed with 3 independent biological replicates (3 technical replicates per sample), and results are presented as mean ± standard deviation (SD). Survival data were analyzed by Kaplan–Meier curves and Log-rank test. Quantitative data (bacterial load, cytokines, intestinal morphology, particle size, stability, and antibacterial activity) were compared using Student’s *t*-test (two groups) or one-way ANOVA followed by Tukey’s posthoc test (multiple groups). Differences were considered significant at *p* < 0.05 and highly significant at *p* < 0.01. Group differences were labeled with lowercase letters (a, b, c), where different letters indicate *p* < 0.05 and the same letter indicates *p* > 0.05. All statistical analyses were conducted using GraphPad Prism 6.0.

## 5. Conclusions

In this study, we successfully developed Lys40-Lip, a cationic liposome-encapsulated bacteriophage endolysin nanoformulation, with favorable physicochemical properties and excellent stability. Via a charge-driven membrane fusion mechanism, Lys40-Lip effectively penetrated the outer membrane barrier of *Salmonella* without additional permeabilizers and exhibited robust bactericidal activity against *Salmonella enteritidis*. In vivo experiments confirmed that Lys40-Lip dose-dependently improved the survival rate of infected chicks, reduced intestinal bacterial colonization, repaired intestinal mucosal damage, and regulated the host inflammatory response. Collectively, Lys40-Lip represents a promising antibiotic alternative for the prevention and control of avian salmonellosis, with high potential for clinical translation in the poultry industry.

## Figures and Tables

**Table 1:** Antibacterial activities of Lys40-Lip toward different species of bacteria.

Strain Type	Phage SP_4 (CFU Reduction, %)	Lys40 (CFU Reduction, %)	Lys40-Lip (CFU Reduction, %)	Empty Liposomes (CFU Reduction, %)
Gram-positive strains				
*Enterococcus faecium* ATCC 29212	ns	82.3 ± 3.1	88.4 ± 1.8	ns
*Staphylococcus aureus* ATCC 29213	ns	85.7 ± 2.8	90.1 ± 2.0	ns
*Streptococcus pneumoniae* ATCC 49619	ns	78.2 ± 3.5	85.6 ± 1.5	ns
Gram-negative strains				
*Salmonella* CVCC 529	ns	ns	86.3 ± 1.6	ns
*Salmonella* ATCC 10426	ns	ns	89.2 ± 1.9	ns
*Salmonella enteritidis* S1	ns	ns	87.1 ± 1.7	ns
*Salmonella enteritidis* S2	72.4 ± 2.9	ns	90.5 ± 2.1	ns
*Salmonella enteritidis* S3	68.1 ± 2.5	ns	87.8 ± 1.6	ns
*Salmonella enteritidis* S4	85.3 ± 3.2	ns	91.2 ± 2.2	ns
*Salmonella enteritidis* S5	ns	ns	84.9 ± 1.4	ns
*Salmonella enteritidis* S6	66.7 ± 2.3	ns	88.7 ± 1.8	ns
*Salmonella enteritidis* S7	73.8 ± 3.0	ns	87.4 ± 1.7	ns
*Salmonella enteritidis* S8	70.2 ± 2.7	ns	89.6 ± 2.0	ns
*Salmonella enteritidis* S9	ns	ns	85.3 ± 1.5	ns
*Salmonella enteritidis* S10	71.5 ± 1.8	ns	88.9 ± 1.9	ns
*Escherichia coli* ATCC 25922	ns	ns	86.7 ± 1.6	ns
*Escherichia coli* ATCC 43895	ns	ns	87.9 ± 1.7	ns
*Escherichia coli* E1	ns	ns	85.2 ± 1.4	ns
*Escherichia coli* E2	ns	ns	89.3 ± 2.0	ns
*Escherichia coli* E3	ns	ns	86.8 ± 1.6	ns
*Escherichia coli* E4	ns	ns	88.1 ± 1.8	ns
*Escherichia coli* O78	ns	ns	90.7 ± 2.1	ns

Data are mean percentage reduction in CFU ± SD from three independent replicates. ns: no significant antibacterial activity, indicating non-significant difference vs. untreated control (*p* > 0.05) or negligible CFU reduction with no bacteriostatic effect.

**Table 2:** Morphometric parameters of intestinal villi in chicks infected with *Salmonella enteritidis* S4 after different treatments.

Intestinal Segment	Parameter	Empty Liposome	Free Lys40	Lys40-Lip-I	Lys40-Lip-II	Lys40-Lip-III	Antibiotic Control	Negative Control	Infected Control
Duodenum	VH (μm)	325.2 ± 18.4 a	342.7 ± 20.1 a	386.5 ± 22.3 b	452.3 ± 25.6 c	468.1 ± 24.8 c	475.6 ± 23.5 c	482.4 ± 21.9 c	298.3 ± 19.7 a
	CD (μm)	182.5 ± 12.1 a	175.3 ± 11.8 a	158.2 ± 10.5 b	132.6 ± 9.2 c	128.4 ± 8.9 c	125.7 ± 9.1 c	122.3 ± 8.7 c	205.6 ± 13.4 a
	VH/CD ratio	1.78 ± 0.12 a	1.95 ± 0.13 a	2.44 ± 0.15 b	3.41 ± 0.18 c	3.64 ± 0.17 c	3.78 ± 0.16 c	3.94 ± 0.15 c	1.45 ± 0.10 a
Jejunum	VH (μm)	302.6 ± 17.5 a	321.4 ± 19.2 a	375.8 ± 21.6 b	442.1 ± 24.3 c	459.3 ± 23.7 c	468.2 ± 22.8 c	476.5 ± 21.2 c	281.5 ± 18.9 a
	CD (μm)	190.3 ± 12.8 a	183.6 ± 12.4 a	162.5 ± 11.2 b	135.7 ± 9.5 c	131.2 ± 9.0 c	128.4 ± 8.8 c	124.6 ± 8.5 c	212.8 ± 14.1 a
	VH/CD ratio	1.59 ± 0.11 a	1.75 ± 0.12 a	2.31 ± 0.14 b	3.26 ± 0.17 c	3.50 ± 0.16 c	3.65 ± 0.15 c	3.82 ± 0.14 c	1.32 ± 0.09 a
Ileum	VH (μm)	285.3 ± 16.8 a	305.7 ± 18.5 a	362.4 ± 20.9 b	428.6 ± 23.1 c	445.2 ± 22.5 c	453.7 ± 21.9 c	462.1 ± 20.8 c	268.9 ± 17.6 a
	CD (μm)	198.5 ± 13.2 a	191.2 ± 12.9 a	168.3 ± 11.6 b	140.5 ± 9.8 c	136.4 ± 9.3 c	133.6 ± 9.2 c	130.2 ± 8.9 c	220.4 ± 14.7 a
	VH/CD ratio	1.44 ± 0.10 a	1.60 ± 0.11 a	2.15 ± 0.13 b	3.05 ± 0.16 c	3.26 ± 0.15 c	3.40 ± 0.14 c	3.55 ± 0.13 c	1.22 ± 0.08 a

Data are presented as mean ± SD (*n* = 6 chicks per group). VH, villus height; CD, crypt depth. Different lowercase letters (a, b, c) indicate significant differences within the same intestinal segment and parameter (*p* < 0.05), analyzed by one-way ANOVA with Tukey’s posthoc test.

**Table 3:** Bacterial species and sources.

Strain Type	Source
Gram-positive strains	
*Enterococcus faecium* ATCC 29212	1
*Staphylococcus aureus* ATCC 29213	1
*Streptococcus pneumoniae* ATCC 49619	1
Gram-negative strains	
*Salmonella* CVCC 529	1
*Salmonella* ATCC 10426	1
*Salmonella enteritidis* S1	3
*Salmonella enteritidis* S2	3
*Salmonella enteritidis* S3	3
*Salmonella enteritidis* S4	2
*Salmonella enteritidis* S5	3
*Salmonella enteritidis* S6	3
*Salmonella enteritidis* S7	3
*Salmonella enteritidis* S8	3
*Salmonella enteritidis* S9	3
*Salmonella enteritidis* S10	3
*Escherichia coli* ATCC 25922	1
*Escherichia coli* ATCC 43895	1
*Escherichia coli* E1	3
*Escherichia coli* E2	3
*Escherichia coli* E3	3
*Escherichia coli* E4	3
*Escherichia coli* O78	2

Note: 1, Purchased from Shanghai Weidi Biotechnology Co., Ltd. (Shanghai, China); 2, Separated and stored in this laboratory; 3, Purchased from China Institute of Veterinary Drug Control (Beijing, China).

**Table 4:** Grouping and treatment of chicks.

Group	Treatment	Infection & Dosing Protocol
A	Empty liposome	Day 5: Oral challenge with 0.5 mL *Salmonella enteritidis S4* (9.2 × 10^9^ CFU/mL); Days 6–8: 0.5 mL empty liposomes daily
B	Free Lys40	Identical infection as Group A; Days 6–8: 0.5 mL free Lys40 (100 μg/mL) daily
C	Lys40-Lip-I	Identical infection as Group A; Days 6–8: 0.5 mL Lys40-Lip
D	Lys40-Lip-II	Identical infection as Group A; Days 6–8: 0.5 mL Lys40-Lip
E	Lys40-Lip-III	Identical infection as Group A; Days 6–8: 0.5 mL Lys40-Lip
F	Antibiotic control	Identical as Group A; Days 6–8: 0.5 mL enrofloxacin (1 mg/mL) daily
G	Negative control	Day 5: 0.5 mL saline; Days 6–8: saline only
H	Infected control	Identical infection as Group A; Days 6–8: saline only

Note: Lys40-Lip-I, Lys40-Lip-II and Lys40-Lip-III: Lys40-loaded liposomes at 100, 200 and 400 μg/mL, respectively.

## Data Availability

The original contributions presented in this study are included in the article. Further inquiries can be directed to the corresponding authors.

## Supplementary Materials

The following supporting information can be downloaded at https://www.mdpi.com/article/10.3390/ani16081193/s1, Figure S1. The result of blastp alignment of Lys40 amino acid; Figure S2. SDS-PAGE analysis of the effects of temperature, IPTG concentration, and induction time on the expression of the recombinant Lys40 fusion protein; Table S1. Lys40-Lip optimal preparation conditions.
