# Supplementary material for: Cationic Liposome-Fused Endolysin Lys40 Overcomes Outer Membrane Barriers and Enhances Survival in Salmonella-Infected Chicks
Source: Animals (Basel). 2026 Apr 14;16(8):1193. doi: 10.3390/ani16081193 (PMC13114078; doi:10.3390/ani16081193)
Supplement: Supplementary file 1 [file animals-16-01193-s001.zip › animals-4200582-supplementary.pdf]

Description	Scientific Name	Max Score	Total Score	Query Cover	E value	Per. Ident	Acc. Len	Accession
lysozyme [Salmonella phage SP_4]	Salmonella phage SP_4	332	332	100%	1e-114	100.00%	162	WKK67930.1
lysozyme [Salmonella phage LPSE1]	Salmonella phage LPS...	331	331	100%	5e-114	99.38%	162	YP_010747884.1
lysozyme [Salmonella phage T102]	Salmonella phage T102	330	330	100%	7e-114	99.38%	162	YP_010748267.1
lysozyme [Salmonella phage PIZ SAE-01E2]	Salmonella phage PIZ...	330	330	100%	2e-113	99.38%	162	QGZ13172.1

Supplementary Figure S1. The result of blastp alignment of Lys40 amino acid

Expression conditions optimization of Lys40 E. coli

By measuring the expression of the target protein under different conditions, the optimal condition was finally selected as 37℃,1 mM IPTG and induction for 5 h with the highest yield (Figure S2).

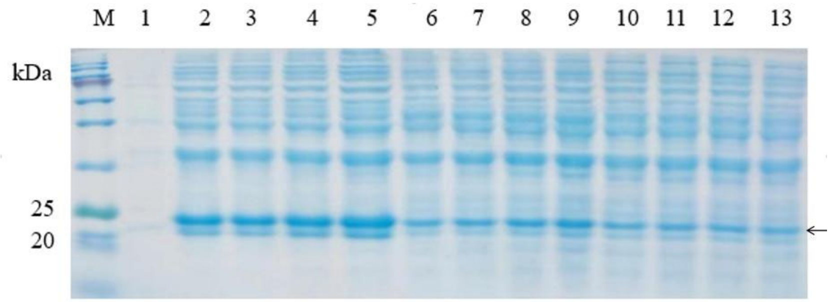

Supplementary Figure S2. SDS-PAGE analysis of the effects of temperature, IPTG concentration, and induction time on the expression of the recombinant Lys40 fusion protein. M, protein molecular weight marker; 1, without IPTG induction; lanes 2-5: 0.125 mM, 0.25 mM, 0.5 mM, and 1 mM IPTG induction at 37°C for 5 h; lanes 6-9: 0.125 mM, 0.25 mM, 0.5 mM, and 1 mM IPTG induction at 28°C for overnight; lanes 10-13: 0.125 mM, 0.25 mM, 0.5 mM, and 1 mM IPTG induction at 16°C for overnight.

Optimal preparation conditions for Lys40-Lip

Single-factor experiments were conducted under fixed parameters (soybean lecithin: 200 mg; hexadecylamine: 33 mg) to evaluate the effects of key variables on encapsulation efficiency (EE). The optimal conditions were identified as follows:

Lecithin/cholesterol mass ratio: 4:1 (EE: 34.49%),

Hydration temperature: 55° C (peak EE: 34.60%),

Hydration time: 120 min (maximum EE: 34.89%),

Sonication power: 20% of total power (1,000 W system, EE: 34.61%),

Sonication duration: 10 min (optimal EE: 34.36%).

Based on the single-factor experimental outcomes, a Box-Behnken design was implemented to optimize three critical parameters—lecithin/cholesterol mass ratio, hydration time, and sonication duration—for maximizing encapsulation efficiency (EE). With EE as the response variable, response surface analysis using Design-Expert software yielded theoretical optimal conditions: a mass ratio of 3.817:1, hydration time of 113.366 min, and sonication duration of 8.343 min. For practical application, these parameters were adjusted to a mass ratio of 3.8:1, hydration time of 113 min, and sonication duration of 8 min 30 s. Under these refined conditions, the encapsulation efficiency reached 33.65% (Table S1).In summary, the optimal formulation was determined as follows: liposomes were prepared using soybean lecithin, cholesterol and hexadecylamine at a mass ratio of 16:4:3.

Table S1
Lys40-Lip optimal preparation conditions

	Mass ratio of soybean lecithin to cholesterol （w/w）	Hydration time （min）	Ultrasonic time （min）	Encapsulation rate(%)
Optimum condition	3.817：1	113.366	8.434	34.83
Adjusted conditions	3.8：1	113	8.5	33.74

Safety Assessment of Lys40-Lip

To evaluate microbial contamination, 100 μL of Lys40-Lip was evenly spread onto LB solid medium and incubated at 37°C for 18 hours, followed by visual inspection for colony formation. Thirty specific pathogen-free (SPF) chicks (purchased from Beijing Melia Verton Laboratory Animal Technology Co., Ltd., Beijing, China) were randomly allocated into two groups (Group A and Group B, n=15 each). Chicks in Group A received oral administration of 500 μL Lys40-Lip (final Lys40 concentration: 100 μg/mL), while those in Group B were administered an equal volume of sterile phosphate-buffered saline (PBS) as a negative control. Clinical signs of morbidity, including abnormal behavior and mortality, were monitored daily throughout the observation period.

Safety Test Results

In Vitro Evaluation

No microbial colonies were observed on LB agar plates after 18 hours of incubation, and the medium surface remained completely transparent, confirming the absence of viable pathogenic bacteria in the Lys40-Lip formulation.

In Vivo Evaluation

All chicks in the Lys40-Lip treatment group exhibited a 100% survival rate, consistent with the control group. No significant abnormalities were detected in feeding behavior, activity levels, or physical appearance throughout the experiment. These findings indicate that Lys40-Lip has no acute toxic effects or pathogenic potential in SPF chicks, supporting its safety for further in vivo applications.
